# PRC2, Chromatin Regulation, and Human Disease: Insights From Molecular Structure and Function

**DOI:** 10.3389/fonc.2022.894585

**Published:** 2022-06-21

**Authors:** Xiuli Liu, Xin Liu

**Affiliations:** Cecil H. and Ida Green Center for Reproductive Biology Sciences, University of Texas Southwestern Medical Center, Dallas, TX, United States

**Keywords:** polycomb-group proteins, PRC2, chromatin, histone methylation, gene regulation, protein structure, developmental disorder, cancer

## Abstract

Polycomb repressive complex 2 (PRC2) is a multisubunit histone-modifying enzyme complex that mediates methylation of histone H3 lysine 27 (H3K27). Trimethylated H3K27 (H3K27me3) is an epigenetic hallmark of gene silencing. PRC2 plays a crucial role in a plethora of fundamental biological processes, and PRC2 dysregulation has been repeatedly implicated in cancers and developmental disorders. Here, we review the current knowledge on mechanisms of cellular regulation of PRC2 function, particularly regarding H3K27 methylation and chromatin targeting. PRC2-related disease mechanisms are also discussed. The mode of action of PRC2 in gene regulation is summarized, which includes competition between H3K27 methylation and acetylation, crosstalk with transcription machinery, and formation of high-order chromatin structure. Recent progress in the structural biology of PRC2 is highlighted from the aspects of complex assembly, enzyme catalysis, and chromatin recruitment, which together provide valuable insights into PRC2 function in close-to-atomic detail. Future studies on the molecular function and structure of PRC2 in the context of native chromatin and in the presence of other regulators like RNAs will continue to deepen our understanding of the stability and plasticity of developmental transcriptional programs broadly impacted by PRC2.

## 1 Introduction

Cell type specification directed by gene expression programs is a central process in the development of multicellular organisms. Gene regulation at the transcriptional level entails a series of complex DNA transactions known to be directly impacted by chromatin architecture. Polycomb repressive complex 2 (PRC2) is a multisubunit chromatin-binding complex, which also harbors intrinsic enzymatic activity responsible for methylation of histone H3 lysine 27 (H3K27) (1–5); trimethylated H3K27 (H3K27me3) is an epigenetic hallmark of gene repression (6). PRC2 plays a vital role in many fundamental biological processes, like genomic imprinting, body segmentation, and vernalization, in various species ranging from humans to flies and plants (7–9). In stem cells, PRC2 cooperates with Polycomb repressive complex 1 (PRC1), which deposits monoubiquitinated H2AK119 (H2AK119ub) (10), to mediate repression of many developmental loci; expression of these loci is necessary for stem cell differentiation (11, 12). Components of both PRC1 and PRC2 belong to the family of Polycomb group (PcG) proteins originally identified in *Drosophila melanogaster* (13). Reversible gene repression marked by H3K27me3 in stem cells predisposes target genes, including tumor suppressor genes, to DNA hypermethylation in cancer cells, which confers permanent gene silencing (14–16). On the other hand, PRC2 functions to maintain cell identity (17); loss of cell identity through dedifferentiation—a mechanism whereby differentiated cells are reverted to progenitor cells—is believed to serve as a mechanism for cancer initiation (18). This review summarizes the current knowledge on the mechanism of PRC2-mediated gene regulation in normal and diseased cells and links molecular function to the recent progress in structural studies of PRC2.

## 2 H3K27 Methylation and PRC2 Targeting

**Figure 1:**
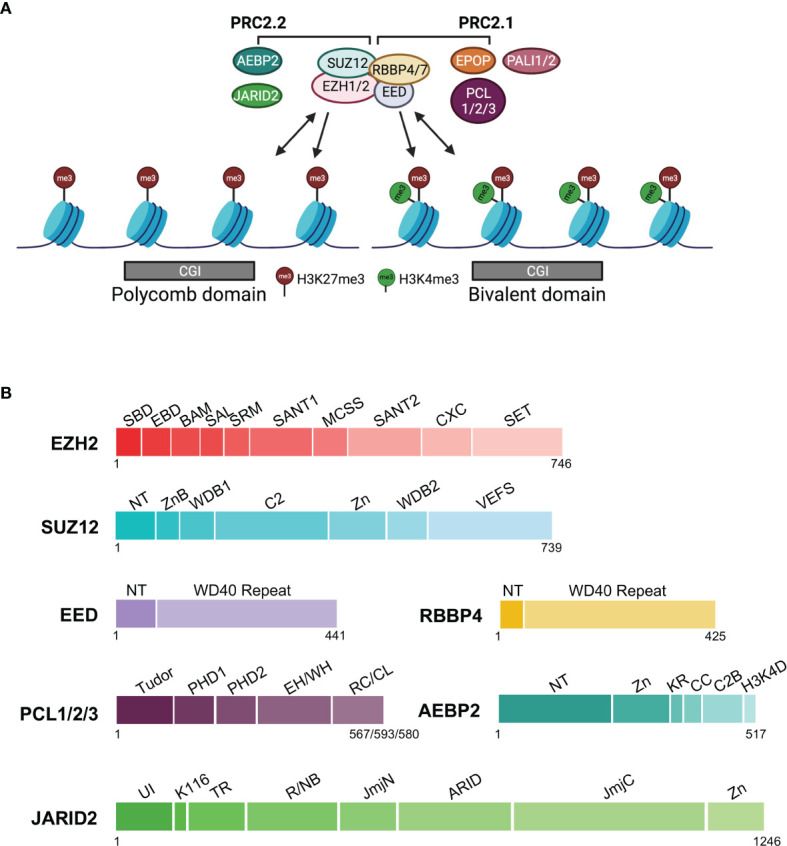
Complex composition, chromatin association, and protein domain structure of PRC2.1 and PRC2.2. The core subunits and accessory subunits specific to PRC2.1 and PRC2.2 are indicated. **(A)** Complex composition and chromatin association. Single-headed arrows depict the deposition of histone marks, and double-headed arrows indicate chromatin binding. CGI chromatin in the Polycomb domain or bivalent domain is represented by gray rectangles. **(B)** Domain structure of PRC2 subunits. EPOP and PALI1/PALI2 of PRC2.1 are not shown, as their 3D structures have not yet been determined. Linker regions are omitted. EZH2: SBD (SANT1-Binding Domain), EBD (EED-Binding Domain), BAM (β-Addition Motif), SAL (SET Activation Loop), SRM (Stimulation-Responsive Motif), SANT1 (SWI3, ADA2, N-COR, and TFIIIB 1), MCSS (Motif Connecting SANT1 and SANT2), SANT2 (SWI3, ADA2, N-COR, and TFIIIB 2), CXC, SET (SU(VAR)3-9, Enhancer-of-zeste and Trithorax); SUZ12: NT (N-Terminal), ZnB (Zinc Finger-Binding), WDB1 (WD40-Binding 1), C2, Zn (Zinc finger), WDB2 (WD40-Binding 2), VEFS (VRN2, EMF2, FIS2, and SU(Z)12); EED: NT (N-Terminal), WD40; RBBP4: NT (N-terminal), WD40; PCL1/2/3 or PHF1/MTF2/PHF19: Tudor, PHD1 (Plant HomeoDomain 1), PHD2 (Plant HomeoDomain 2), EH/WH (Extended Homology/Winged-Helix), RC/CL (Reversed Chromo/Chromo-Like); AEBP2: NT (N-terminal), Zn (Zn finger), KR (K (lysine) and R (arginine)-rich), CC (Central Connecting), C2B (C2-Binding), H3K4D (H3K4 Displacement); JARID2: UI (Ubiquitin Interaction), K116 (K (lysine) 116), TR (TransRepression), R/NB (RNA/Nucleosome Binding, JmjN (Jumonji N), ARID (AT-Rich Interaction Domain), JmjC (Jumonji C), Zn (Zinc finger).

PRC2 impacts gene expression at the transcriptional level in large part through H3K27 methylation (**Figure 1A**). The H3K27me3 repressive histone mark is enriched in Polycomb domains in distinct genomic regions (**Figure 1A**), such as promoters of inactive or lowly expressed genes, intergenic regions, subtelomeric regions, transposable elements, and megabase domains on the X chromosome (6, 19, 20). In human and mouse embryonic stem cells (hESCs and mESCs), H3K27me3 is often colocalized with the H3K4me3 active histone mark, which defines ‘bivalent’ chromatin domains poised for gene activation (**Figure 1A**) (21–25). Other histone marks associated with gene silencing include H2AK119ub, H3K9me3, and H4K20me3. In contrast to frequent colocalization of H2AK119ub and H3K27me3 at facultative heterochromatin, H3K9me3 and H4K20me3 are typical histone marks associated with constitutive heterochromatin (26, 27). Mutual exclusivity of H3K27me3 and H3K9me3 is not strictly maintained in some contexts (20). In addition to trimethylation, PRC2 also mediates mono and dimethylation of H3K27; whereas more than 50% of total histone H3 is dimethylated at K27 (H3K27me2), H3K27me3 and monomethylated H3K27 (H3K27me1) are substantially less abundant at about 10–20% in mESCs (27–29). Notably, H3K27me1 accumulates at gene bodies of highly transcribed loci (29).

PRC2 is preferably targeted to CpG islands (CGIs) in cells (**Figure 1A**) (25, 30). At the same time, binding sites of many transcription factors are also located in GC-rich CGI promoters (31, 32). Hypomethylated CGIs lacking transcription factor binding and active transcription is sufficient to recruit PRC2 (33–35). In *Drosophila*, PRC2 is targeted to chromatin *via* DNA motifs known as Polycomb response elements (PREs) and cognate transcription factors; in comparison, how the chromatin recruitment of mammalian PRC2 is achieved remains incompletely understood, primarily due to the lack of conservation of PREs in mammals (36–38). Unlike transcription factors that initiate transcription on specific genes, PRC2 does not trigger gene repression; instead, PRC2 targeting and H3K27me3 deposition occur after transcriptional silencing (39, 40). Accordingly, the primary function of PRC2 is thought to maintain established gene expression programs corresponding to discrete cell types (17). PRC2 is dispensable for pluripotency and self-renewal of mESCs but is required for proper stem cell differentiation (41, 42). For example, the formation of embryoid bodies (EBs) through mESC differentiation is impaired when all three H3K27 methylation states are reduced, which coincides with an increase of the acetylated H3K27 (H3K27ac) active histone mark (43). In contrast, EB formation is not affected when only H3K27me3 but not H3K27me1 or H3K27me2 is markedly diminished (43, 44). Prominently, the differentiated cell identity is reverted to a stem cell-like state when single cells dissociated from EBs are challenged to grow in stem cell media in the absence of full H3K27 trimethylation, highlighting an essential role of PRC2 and H3K27me3 in the maintenance of differentiated cell identity (44).

## 3 Core and Accessory Subunits of PRC2

A functional mammalian PRC2 holo complex consists of core and accessory subunits (**Figures 1A, B**). EZH2 or its paralog EZH1 is the catalytic subunit, each containing a SET (SU(VAR)3-9, Enhancer-of-zeste and Trithorax) lysine methyltransferase domain (**Figures 1A, B**) (45–48). Other core subunits include EED, SUZ12, and another pair of paralogs, RBBP4 and RBBP7 (**Figures 1A, B**). EED and SUZ12 are necessary for PRC2 enzymatic activity, whereas RBBP4 substantially enhances catalysis by the EZH2–EED–SUZ12 ternary complex (49). Knockout of EZH2, EED, or SUZ12 causes embryonic lethality in mice (50). EED recognizes H3K27me3, a product of PRC2 catalysis, leading to the allosteric stimulation of PRC2 enzymatic activity. This positive feedback mechanism is believed to at least partially account for the spreading of the H3K27me3 repressive histone mark in large chromatin domains (51, 52).

Biochemistry and proteomics studies have identified different families of accessory subunits, which form two classes of PRC2 holo complexes, PRC2.1 and PRC2.2, based on their mutually exclusive binding to the core subunits (**Figures 1A, B**) (53, 54). PHF1/MTF2/PHF19, EPOP, and PALI1/PALI2 belong to PRC2.1, whereas AEBP2 and JARID2 are present in PRC2.2 (**Figures 1A, B**). PHF1 (a.k.a. PCL1), MTF2 (a.k.a. PCL2), and PHF19 (a.k.a. PCL3) are three homologs of the *Drosophila* PCL protein, and among them MTF2 is the most abundant in mESCs. In the absence of AEBP2, MTF2 and JARID2 can form an atypical hybrid holo complex with the core subunits (53). PRC2.1 and PRC2.2 colocalize at most PRC2 target loci in mESCs; combined knockout of PHF1/MTF2/PHF19 and JARID2 disturbs PRC2 targeting and results in diffusely distributed H3K27me3 on chromatin, highlighting a pivotal role of these accessory subunits in the locus-specific chromatin recruitment of PRC2 (55, 56). In human induced pluripotent stem cells (hiPSCs), selective disruption of PRC2.1 favors PRC2.2 complex formation, and vice versa; when PRC2.2 is disrupted and PRC2.1 is forced to form, overall chromatin occupancy of PRC2 is increased, likely due to a higher chromatin binding affinity of PRC2.1 compared to that of PRC2.2 (57). Despite the largely overlapped chromatin association of PRC2.1 and PRC2.2, they regulate mESC differentiation *via* distinct mechanisms: MTF2-containing PRC2.1 maintains the silent state of target genes already marked by H3K27me3 in mESCs, whereas JARID2-containing PRC2.2 preferentially mediates *de novo* repression of active genes (58). Existing data indicate that the mechanism of how accessory subunits facilitate PRC2 recruitment involves their direct binding to linker DNAs and histone marks (see 6.3 below).

## 4 PRC2 Dysregulation in Human Disease

Dysregulation of PRC2 function is widely associated with human diseases, including cancers and developmental disorders. Hotspots of mutation include EZH2 residues from the catalytic SET domain and a structural motif mediating the allosteric stimulation of PRC2 (see 6.2 below) (59). Both gain-of-function and loss-of-function changes of PRC2 are linked to cancer, which reflects contradicting roles of PRC2 in oncogenesis and tumor suppression (17, 60). For example, H3K27 hypertrimethylation required for the growth of a subset of human B-cell lymphoma is generated by heterozygous activating mutations of EZH2 (61–64). In addition, cancer dependence on EZH2 sometimes relies on genetic alterations of the SWI/SNF chromatin remodeling complex, making EZH2 an epigenetic target for drugs based on synthetic lethality (65, 66). Furthermore, EZH2 expression is correlated with active cell proliferation, and EZH2 is often aberrantly overexpressed in a number of cancer types, such as breast cancer, prostate cancer, endometrial cancer, melanoma, glioblastoma, ovarian cancer, lung cancer, and so on, where tumor suppressor genes, DNA damage repair genes, and cell signaling genes are among the targets of PRC2 (17, 67–69). On the other hand, an elevated EZH2 expression level may merely be a consequence of tumorigenesis in some cases (65). Pharmacological inhibition of PRC2 enzymatic activity by a panel of clinically relevant specific pyridone inhibitors markedly restricts the proliferation of PRC2-dependent cancer cells (68, 70).

In line with its role in tumor suppression, loss-of-function of PRC2 caused by inactivating mutations or chromosomal translocation of essential components of PRC2, including EZH2, EED, and SUZ12, is frequently found in myelodysplastic syndrome/myeloproliferative neoplasm (MDS/MPN), T cell acute lymphoblastic leukemia (T-ALL), and malignant peripheral nerve sheath tumors (MPNSTs) (17, 68). Notably, PRC2 is enzymatically inactivated by protein factors in distinct types of cancers, including diffuse midline gliomas expressing oncohistone H3 with a K27M mutation (H3K27M) and posterior fossa ependymoma expressing EZHIP that harbors an H3K27M-mimicking sequence (71–77). Likewise, genetic mutations of PRC2 subunits and especially EZH2 have been found to impair H3K27 trimethylation and cause Weaver syndrome and Cohen-Gibson syndrome, which are both overgrowth syndrome often characterized by facial deformity and intellectual disability (78).

PRC2 is also connected to cancer *via* cell signaling and metabolic pathways. For example, EZH2 phosphorylation by the AKT kinase suppresses PRC2 enzymatic activity and promotes hormone-refractory prostate cancer independent of other PRC2 subunits (79–81). In addition, EZH2 is phosphorylated by AMP-activated protein kinase (AMPK) upon energy starvation, which leads to PRC2 disassembly, cause derepression of tumor suppressor genes, and correlates with better survival in cancer patients (82). The intracellular concentrations of SAM and SAH profoundly impact histone methylation (83, 84). SAM is synthesized from methionine and ATP by methionine adenosyltransferase (MAT), and methionine restriction reduces the H3K27me3 level in HCT116 colon cancer cells and C57BL/6J mice (84). Conversely, DZNep, an SAH hydrolase inhibitor, also inhibits H3K27 methylation and induces apoptosis in breast cancer cells by reactivating certain apoptosis factors (85). Some more details of the PRC2–disease connection are further discussed below where PRC2 structures are analyzed.

## 5 Mode of Action of PRC2 in Gene Regulation

Several direct or indirect mechanisms have been proposed to underlie PRC2-mediated transcriptional regulation. Listed below are emerging models involving key aspects of the molecular function of PRC2, such as enzymatic activity, RNA and chromatin binding, and protein-protein interaction. Active transcription is kept in check from multiple levels, ranging from antagonization of deposition of active histone marks to crosstalk with components of RNA polymerase II (pol II) transcription machinery and to orchestration of high-order chromatin structure.

### 5.1 Competition Between H3K27 Methylation and Acetylation

**Figure 2:**
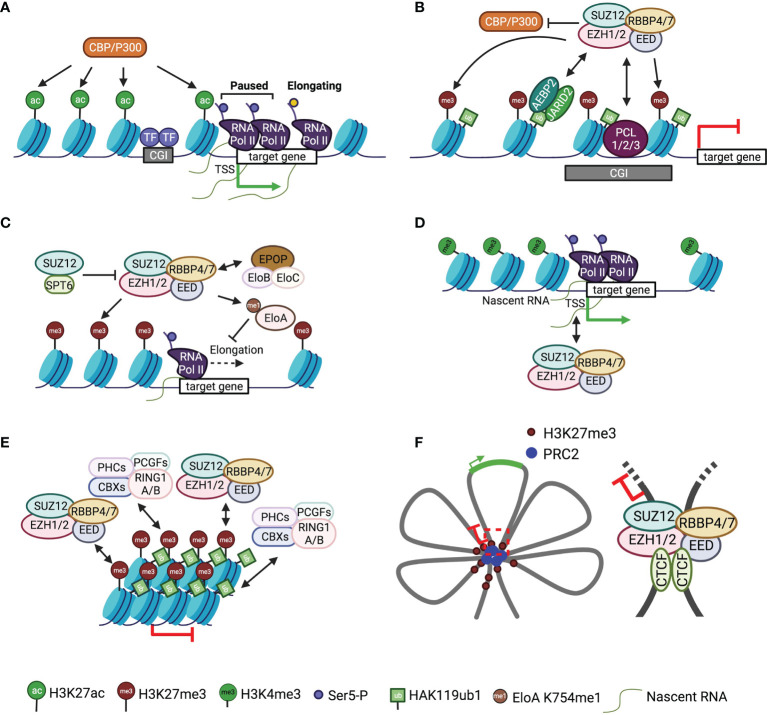
Mode of action of PRC2 in gene regulation A schematic of the mode of action of PRC2 is shown. Single-headed arrows depict the deposition of histone marks, and double-headed arrows indicate chromatin or RNA binding. **(A)** A simplified version of the schematic of typical active gene loci. **(B)** Antagonistic action of PRC2 and P300/CBP in histone H3K27 modification. **(C)** Crosstalk of PRC2 with transcription elongation factors. **(D)** Interaction of PRC2 with nascent RNAs. **(E)** Chromatin compaction by PRC2 and PRC1. **(F)** Left, schematic of multiple connected chromatin loops with repressed and active loci; right, a representative chromatin loop bound by CTCF and PRC2.

Lysine acetylation of histone proteins usually correlates with active transcription, whereas lysine methylation can mark either gene activation or repression depending on positions of the lysine residue and methylation multiplicity (86, 87). H3K27 acetylation is mediated by p300/CBP histone acetyltransferase (HAT), and H3K27ac is preferentially enriched at both promoters and active enhancers (88, 89) (**Figure 2A**). Studies in *Drosophila* embryos and mESCs have shown that H3K27ac and H3K27me3 are antagonistic, suggesting a direct competition between PRC2 and p300/CBP in posttranslational modification of H3K27 (**Figure 2B**) (90, 91). Furthermore, PC, a component of *Drosophila* PRC1, impedes H3K27 acetylation by directly inhibiting the HAT activity of CBP (92). In line with the competition model, the widespread deposition of H3K27me2 is believed to protect target loci from HAT activity, ensuring proper control of cell type-specific enhancers (29). Additionally, EZH1 or a catalytically impaired EZH2 mutant is unable to fully restore H3K27me2 or H3K27me3 levels in an EZH2 knockout background, leading to invasion of H3K27ac into the otherwise H3K27me3-marked promoters and causing defects in mESC differentiation (43).

### 5.2 Crosstalk With Transcription Machinery

PRC2 is mechanistically linked to pol II transcription elongation through Elongin and SPT6 (**Figure 2C**). Elongin positively regulates pol II elongation by suppressing transcriptional pausing; the heterotrimeric Elongin complex consists of the largest active subunit Elongin A and two smaller regulatory subunits, Elongin B and C (93–95). PRC2 methylates Elongin A at residue K754, and loss of this methylation results in de-repression of PRC2 target genes and defective EB formation (**Figure 2C**) (96). In addition, the accessory subunit EPOP from PRC2.1 has been proposed to connect Elongin B and C to PRC2 in mESCs, maintaining low expression of PRC2 target genes through a mechanism that is not understood (**Figure 2C**) (97, 98). Direct crosstalk also exists between PRC2 and the elongation factor SPT6: PRC2 recruitment and H3K27me3 deposition are impeded by SPT6 due to the competitive binding of SPT6 and EZH2 to SUZ12, which blocks the assembly of functional PRC2 (**Figure 2C**) (99).

RNAs provide another link between PRC2 and transcription machinery (**Figure 2D**). PRC2 mediates promiscuous binding to RNAs with a preference for G-tract sequences (100, 101). RNAs associate with dispersed sites on PRC2 surface (102, 103), antagonizing direct chromatin binding of PRC2 but linking PRC2 to chromatin physically (104, 105). In particular, PRC2 interacts with short RNAs transcribed from target genes, where pol II and H3K4me3 are observed, to repress gene expression *in cis* in primary T cells (**Figure 2D**) (106). Congruently, PRC2 is also found to bind nascent RNAs from active promoters devoid of H3K27me3 in mESCs (**Figure 2D**) (107). RNAs can inhibit PRC2 enzymatic activity both *in vitro* and *in vivo*, possibly by preventing nucleosomal substrate binding (108–110), which may serve as a mechanism to sustain the active state of genes while keeping PRC2 poised for chromatin surveillance (100, 107, 109). Additional data suggest PRC2 interaction with nascent transcripts can tune the transition between promoter-proximal pausing and productive elongation (**Figures 2A, D**) (111).

### 5.3 Formation of High-Order Chromatin Structure

PRC2 has been linked to at least two types of distinct high-order chromatin structures: compacted chromatin and long-range chromatin loops or contacts. Chromatin compaction causes transcriptional inactivation by restricting promoter accessibility (**Figure 2E**). In a hierarchical recruitment model, PRC2 is targeted to chromatin decorated by H3K27me3, which recruits canonical PRC1 *via* the chromodomain (CD) of CBX proteins (112). This allows PHC and CBX2 subunits of PRC1 to compact target chromatin, independent of PRC1 enzymatic activity (113–116). EZH1-containing PRC2 also exhibits chromatin compaction activity, which appears to depend on histone tails but is independent of SAM (67). Unlike the EZH2-containing counterpart, EZH1-containing PRC2 displays a higher binding affinity towards nucleosomes and, correspondingly, represses *in vitro* transcription from chromatinized templates (67, 117).

Long-range chromatin interactions between genes and regulatory elements, like enhancers, repressors, and insulators, are widely implicated in gene regulation in the context of a three-dimensional genome, for which PRC2 and H3K27me3 are also known as important players (118–123). H3K27me3-rich genomic regions can act as gene silencers through PRC2-dependent long-range chromatin interactions (124, 125). Differential chromatin binding of PRC2 along the HoxA cluster impacts long-range interactions of HoxA in mouse embryos, which unexpectedly also promotes enhancer-promoter contacts (126). Likewise, PRC2 facilitates contact between poised enhancers and target genes during mESC differentiation (127). Hox clusters are organized into multiple connected chromatin loops, which involve PcG proteins and other architectural factors, like CTCF (**Figure 2F**) (128, 129). Relatedly, the SUZ12 subunit of PRC2 has been found to interact with CTCF at a long-range intrachromosomal loop between IGF2/H19 imprinting control region (ICR) and IGF2 promoters, maintaining monoallelic expression of IGF2 (**Figure 2F**) (130, 131). In line with these data, PRC2 directly mediates DNA bending and looping in reconstituted systems, as shown by atomic force microscopy (AFM) and molecular dynamics simulations (132, 133).

## 6 Structural Mechanism of PRC2 Function

Over the years, the structural biology of PRC2 has generated a large body of knowledge on the molecular mechanism of some central aspects of PRC2 function, including complex assembly, enzyme catalysis, and chromatin recruitment, which are known to be dysregulated in diverse human diseases. First, structures of PRC2 subcomplexes and holo complexes have together shed light on the overall architecture of PRC2 and have provided a structural basis for the mutually exclusive assembly of PRC2.1 and PRC2.2. Second, the catalytic mechanism has been elucidated from the structure of an active PRC2, which also lays the foundation for a mechanistic understanding of cancer mutations, drug inhibition, oncohistone-mediated enzyme inactivation, and allosteric stimulation of PRC2. Third, structures of PRC2 engaging with nucleosomes and of functional domains bound to linker DNAs or histone marks have offered direct visualization of PRC2 targeting in various chromatin contexts.

### 6.1 Complex Assembly

**Figure 3:**
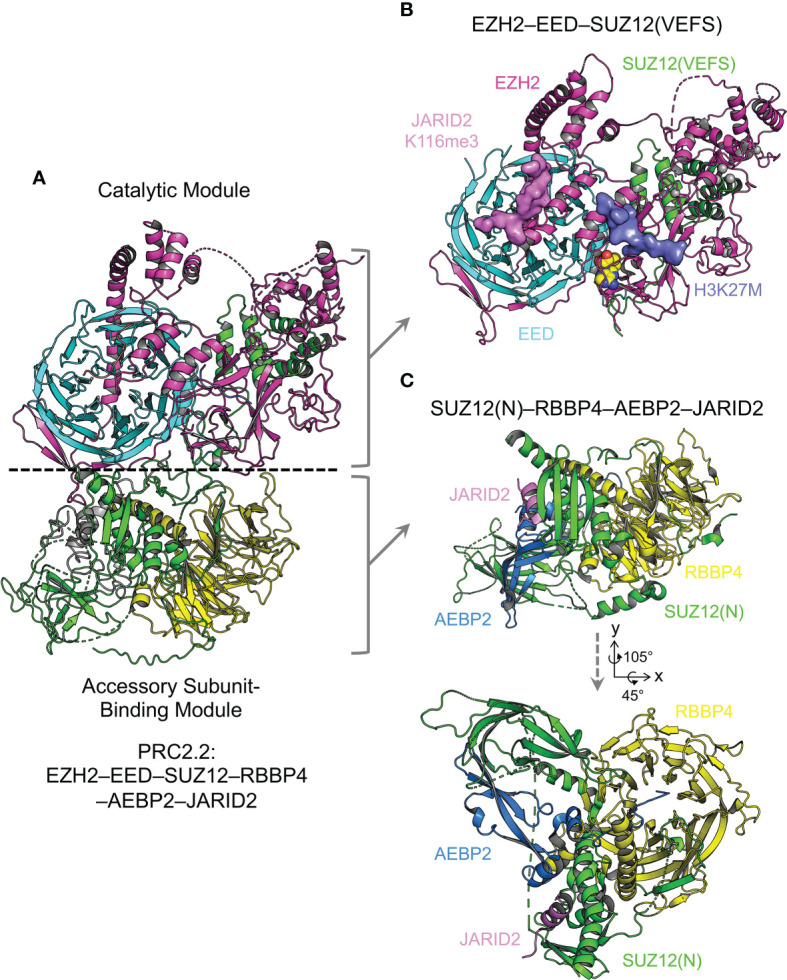
Overall structure of PRC2.2 The overall structure of PRC2 is revealed by X-ray crystallographic and cryo-EM studies. The PRC2 core complex can be structurally divided into two modules. Components of structures are color-coded. Structure figures are rendered in PyMOL (The PyMOL Molecular Graphics System, Version 2.5.2 Schrödinger, LLC). **(A)** Cryo-EM structure of AEBP2 and JARID2-bound PRC2.2 (PDB 6C23). **(B)** Crystal structure of the catalytic module of PRC2 (PDB 5HYN). H3K27M and JARID2K116me3 peptides are shown as surface representation. **(C)** Crystal structure of the accessory subunit-binding module bound to AEBP2 and JARID2 (PDB 5WAI). Two views are shown with the rotation matrix indicated.

Proper assembly of core and accessory subunits is a prerequisite for PRC2 enzymatic activity and chromatin recruitment. Crystal structures of a minimal mouse EZH2–EED complex and of NURF55 (homolog of RBBP4 in *Drosophila*) bound to a minimal fragment of SU(Z)12 (homolog of SUZ12 in *Drosophila*) represent the initial attempts to tackle the puzzle of complex assembly (134, 135). The negative stain EM structure of a five-member PRC2.2, PRC2-AEBP2, further reveals a bipartite structure architecture (136). Recent X-ray crystallographic and cryo-EM studies have together built a near-complete atomic model of the core complex and started to add pieces of various accessory subunits into the picture of holo complex structures (**Figure 3**). Overall, the PRC2 core complex consists of two structurally and functionally distinct modules, including the catalytic module and the accessory subunit-binding module (**Figure 3A**) (137): whereas the former contains the minimally active ternary complex of EZH2, EED, and the C-terminal VEFS (VRN2, EMF2, FIS2, and SU(Z)12) domain of SUZ12 (SUZ12(VEFS)) (**Figure 3B**) (138–140), the latter is composed of the N-terminal portion of SUZ12 (SUZ12(N)) and RBBP4, which together form docking sites for various accessory subunits (**Figure 3C**) (140, 141). EZH2 is folded into a series of functional domains dispersed across the catalytic module (138, 139) (**Figure 3B**). EED and SUZ12(VEFS) are bound to distinct subsets of EZH2 domains, juxtaposing with each other (**Figure 3B**) (138, 139). In the accessory subunit-binding module, functional domains of SUZ12(N) are scattered on the surface of the RBBP4 WD40 repeat, forming both intramolecular and intermolecular complexes (**Figure 3C**) (141, 142). The accessory subunit-binding module also mediates PRC2 dimerization in the context of PRC2.1, which facilitates chromatin binding of PRC2 (see below) (142). Key structural features of the assembly of these two modules are described in sections below, with the disease connection or clinical relevance highlighted.

**Figure 4:**
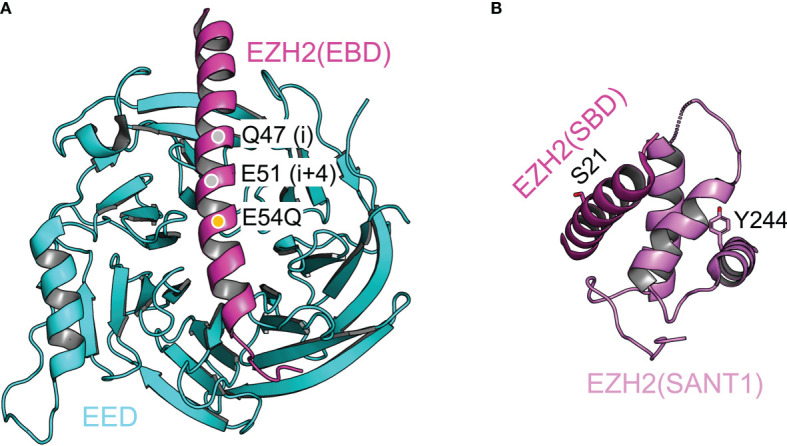
Structural dissection of the complex assembly of the catalytic module Structural features of the catalytic module are highlighted. **(A)** Structure of the EZH2(EBD)–EED complex (from PDB 5HYN). Q47 and E51 (gray discs) are the two residues at positions i and i+4, where a hydrocarbon staple is incorporated. E54Q mutation (orange disc) is introduced to enhance the cellular uptake of the stapled peptide. **(B)** Intramolecular complex of EZH2(SBD) and EZH2(SANT1). Residues S21 and Y244, which are phosphorylated in cancer cells, are labeled and shown as sticks representation.

*EZH2–EED interaction and stapled peptide inhibitor of PRC2.* In the catalytic module of PRC2, the EBD (EED-binding domain) of EZH2 associates with the bottom face of the EED WD40 repeat, dominating the interaction of these two proteins (**Figure 4A**) (134, 138, 139). Stapled α-helical peptide inhibitors have opened a new window for drug discovery by targeting protein-protein interactions, which has been difficult with small molecule inhibitors (143). Stapled peptides designed based on the EBD structure compete with EZH2 for EED binding *in vivo*, leading to disruption of PRC2 assembly (**Figure 4A**) (144). These peptides inhibit PRC2-dependent growth and induce differentiation of MLL-AF9 leukemia cells (144).

*Intramolecular complex and gene activation function of EZH2 in cancer cells.* In the catalytic module of PRC2, the SANT1 (SWI3, ADA2, N-CoR, and TFIIIB 1) domain and the SBD (SANT1-binding domain) of EZH2 mediate intramolecular interactions (**Figure 4B**) (138, 139). The SANT1 harbors a partially disordered acidic transactivation domain (TAD), which is normally sequestered by SBD binding (145). The TAD can be released by EZH2 phosphorylation at residue S21 by AKT kinase or at residue Y244 by JAK3 kinase (**Figure 4B**) (145). These two phosphorylation events are activated in prostate cancer and natural killer/T-cell lymphoma cells, respectively, converting EZH2 from a gene repressor to a gene activator (80, 81, 146). In line with this model, the EZH2 TAD forms stable complexes with components of the active transcription machinery, such as p300/CBP (145). Intriguingly, the EZH2 TAD interacts with the MYC oncoprotein in a PRC2-independent manner to promote the growth of leukemia cells (147). Indeed, EZH2 also physically associates with MYC or MYCN in other cellular contexts, including neuroblastoma and prostate cancer cells, to drive cancer development, making EZH2 degradation an attractive strategy for cancer treatment (147–150). In contrast, it remains to be shown whether enzymatic inhibitors of PRC2 can specifically target the ‘solo’ EZH2, as the drug-binding site may not exist in the absence of EED and SUZ12 (see **6.2** below).

**Figure 5:**
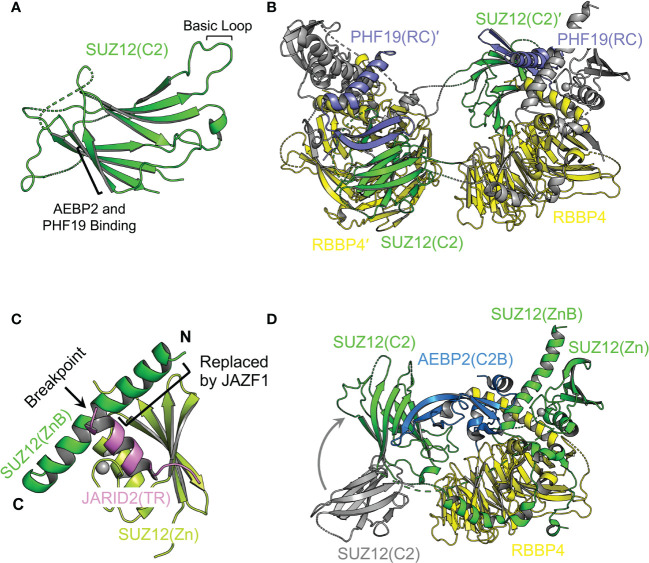
Structural dissection of the complex assembly of the accessory subunit-binding module Structural features of the accessory subunit-binding module are highlighted. **(A)** Structure of the SUZ12(C2) domain (from PDB 5WAI). The basic loop for RBBP4 binding and β strand for ABEP2 and PHF19 binding are indicated. **(B)** Structure of the SUZ12(N)–RBBP4 dimer stabilized by the PHF19(RC) domain (from PDB 6NQ3). SUZ12 is colored in gray except for the C2 domain, which is swapped between two protomers and shown in green. Domains and subunits from two protomers are distinguished by the prime symbol. **(C)** Structure of AEBP2 bound to SUZ12(N)–RBBP4 (from PDB 5WAI). The C2 domain from the dimeric structure is shown in gray. Structural transition of the C2 domain accompanied by AEBP2 binding and dimer disruption is indicated by a curved gray arrow. The JARID2(TR) is omitted for clarity. **(D)** Structure of JARID2(TR) bound to the intramolecular complex of SUZ12(ZnB) and SUZ12(Zn) (from PDB 5WAI). The breakpoint of SUZ12(ZnB) in oncogenic chromosomal translocation is indicated by a black arrow. The black bracket indicates the part of the SUZ12(ZnB) helix replaced by JAZF1.

*C2 domain, a mediator of mutual exclusivity and PRC2 dimerization.* In the accessory subunit-binding module of PRC2, SUZ12(N) contains a mobile non-canonical C2 domain, which becomes ordered only when bound to the accessory subunit PHF19 or AEBP2 (**Figure 5A**) (141, 142). The C2 domain is named based on the observation that it adopts an eight-strand β-sandwich structure, mimicking the classical C2 domain that mediates phospholipid binding (**Figure 5A**) (141). It is not unprecedented that phospholipids regulate the function of chromatin complexes, and it remains to be explored if such a mechanism also exists for PRC2 (141, 151). PHF19 and AEBP2 interact with overlapping surfaces of the C2 domain, which accounts for the mutual exclusivity in complex assembly between PRC2.1 and PRC2.2 (141, 142). Formation of a PRC2 dimer has long been implicated (67, 117, 152–155), and recent structural work reveals that the PRC2 core complex forms an intrinsic dimer *via* the C2 domain of SUZ12: a basic loop region on the C2 domain from one protomer is swapped to occupy the acidic central cavity of RBBP4 from the other protomer (**Figures 5A, B**) (142). This dimeric structural scaffold has a profound impact on the ability of PRC2 to bind chromatin (see below).

*PHF19 binding and stabilization of the intrinsic PRC2 dimer.* The RC (reversed chromo) domain of PHF19 (PHF19(RC)) mediates concurrent binding to the C2 domain of SUZ12 from one protomer and the SUZ12(N)–RBBP4 protein body from the other protomer (142) (**Figure 5B**). In this way, PHF19 is not involved in PRC2 dimerization *per se*; instead, it stabilizes the intrinsic dimer of PRC2 (**Figure 5B**). Structure-guided functional studies indicate that PRC2 dimerization promotes chromatin binding of MTF2 or PHF19-containing PRC2.1, possibly through an avidity effect (142). There is no evidence on whether PRC2 plays a direct architectural role in forming long-range chromatin contacts. However, it is not impossible that the dimeric structural scaffold may allow PRC2 to bring two distal chromatin regions close to each other *via* direct chromatin binding. In this regard, a set of specific structure-based dimer-disrupting mutations can serve as a valuable tool to test this possibility (142).

*AEBP2 binding and the dimer-to-monomer transition.* There are long and short isoforms of AEBP2 in mammals (156). AEBP2 used in the published structural studies corresponds to the isoform 2 (UniProtKB Q6ZN18-2), lacking the N-terminal D/E-rich, S-rich, and G-rich patches (140, 141, 157). The C2B (C2-binding) domain of AEBP2 (AEBP2(C2B)) is bound to the C2 domain of SUZ12 (**Figure 5C**) (141). A part of the AEBP2(C2B) also associates with the ZnB (zinc finger binding) helix of SUZ12 (SUZ12(ZnB)), where disease mutations are found to specifically disrupt PRC2.2 assembly (**Figure 5C**) (57, 141). Notably, AEBP2 binding induces a dimer-to-monomer transition of the PRC2 core complex by relocating the swapped C2 domain of SUZ12, suggestive of drastically different structural architectures of PRC2.1 and PRC2.2 (142).

*JARID2 binding, mutual exclusivity, and oncogenic chromosomal translocation.* The Zn (zinc finger) domain and ZnB helix of SUZ12 assemble into a binding platform for the TR domain of JARID2 (JARID2(TR)) (**Figure 5D**) (141). An overlapping surface may also mediate EPOP binding according to biochemical data, underlying the mutual exclusivity between JARID2 and EPOP in the assembly of PRC2.1 and PRC2.2 holo complexes (141). The N-terminal half of the SUZ12(ZnB) helix is replaced by the zinc finger protein JAZF1 in a recurrent oncogenic chromosomal translocation found in endometrial stromal tumors (**Figure 5D**) (158). In line with the structural finding that the SUZ12(ZnB) helix is essential for JARID2 binding, PRC2 containing the JAZF1-SUZ12 fusion protein displays greatly impaired JARID2 association, which may at least in part account for the global loss of the H3K27me3 histone mark in patient samples (141, 159).

### 6.2 Enzyme Catalysis

**Figure 6:**
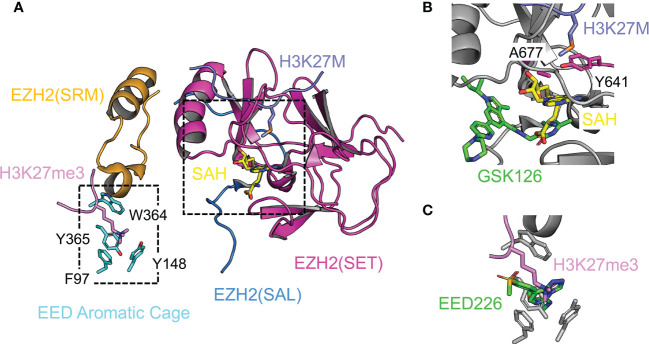
Structural analyses of enzyme catalysis, chemical inhibition, and protein inhibition Enzyme active site is shown. **(A)** Structure of the catalytic module of PRC2 in the stimulated state (from PDB 5HYN). The split catalytic domain consists of the EZH2(SET) and EZH2(SAL). The EZH2(SRM) bridges the stimulating signal to the EZH2(SET). EED aromatic cage residues are labeled. H3K27M oncohistone occupies the lysine binding channel at the active site. The JARID2K116me3 peptide in the original structure was replaced by the H3K27me3 peptide from PDB 3IIW based on the structural alignment. **(B)** Close-up view of the active site. SAM-competitive PRC2 inhibitor GSK126 from the aligned crystal structure PDB 5WG6 is represented by green sticks. **(C)** Close-up view of the EED aromatic cage. The allosteric PRC2 inhibitor EED226 from the aligned crystal structure PDB 5GSA is represented by green sticks.

EZH1 and EZH2 represent a distinct lysine methyltransferase family (160). The SET domains of EZH1 and EZH2 differ from many other SET domains by requiring two binding partners, EED and SUZ12, for the enzymatic activity (49). Structural data indicate that EZH2 contains an unusual split catalytic domain: the SAL (SET activation loop) from the N-terminal portion and the SET domain at the C-terminus are folded together to enable catalysis (**Figure 6A**) (138, 139). Structural comparison to the inactive conformation of an isolated SET domain of EZH2 indicates that the SET domain undergoes structural reorganization from the inactive to the active state, reshaping the H3-binding groove and SAM-binding pocket for effective substrate binding (138, 139, 161, 162). H3K27me3 is a major mediator of PRC2 function in gene regulation in cells; correspondingly, the enzymatic activity is a focal point of the cellular regulation of PRC2 function. The catalytic mechanism, enzyme regulation in normal cells, and enzyme dysregulation in diseased states are summarized in the following paragraphs.

*The catalytic mechanism, cancer mutations, and SAM-competitive inhibitors*. Histone substrate recognition and methyl transfer reaction are two key steps for PRC2-mediated H3K27 methylation. The A25-R26-K27-S28 core sequence within the histone H3 tail and in particular residue R26 are read by the SET domain directly; residue A25 also contributes to the substrate specificity by the mechanism of steric exclusion (139). Enzyme reactivity data on *in vitro* methylation of peptide arrays have expanded the substrate specificity of PRC2 to a preferred sequence of [A/C/V/P]24-[A/V/L]25-[R/K]26-[K]27-[F/Y/H]28-[A/V/C/T/S]29 (96). Phosphorylation of residue S28 of histone H3 by cell signaling-dependent MSK kinases diminishes H3K27 methylation, displaces PRC2 from chromatin, and promotes H3K27 acetylation (96, 163). Based on the structure of PRC2 bound to H3K27M (**Figure 6A**), the substrate residue K27 is predicted to be hosted in the lysine-binding channel lined by several aromatic residues such that its nitrogen atom is placed close to SAM for the methyl transfer reaction (138, 139). Compared to H3K27me1 and H3K27me2, the production of H3K27me3 is naturally disfavored because of spatial restriction at the active site (139). Residue Y641 of EZH2 at the enzyme active site is mutated to smaller residues, like F, N, S, H, or C, in follicular and diffuse large B-cell lymphomas, which results in an H3K27 hypertrimethylation disease phenotype, possibly due to a relatively expanded active site (**Figure 6B**) (61–63). Another gain-of-function active site mutation, A677G, found in human B-cell lymphoma, causes hypertrimethylation likely *via* a similar structural mechanism (**Figure 6B**) (64). A family of SAM-competitive pyridone inhibitors of EZH2, like GSK126, EPZ-6438, and CPI-1205, have been developed to treat cancers with EZH2 gain-of-function mutations as well as other EZH2-dependent cancers (164). These inhibitors exhibit high potency and specificity and are mostly bound to a binding pocket within a unique interface between the SAL and SET of EZH2, with only a small set of atoms protruding into the SAM binding pocket to block SAM binding (**Figure 6B**) (165–167).

*Allosteric stimulation, Weaver syndrome mutations, and H3K27me3-competitive inhibitors.* H3K27me3 stimulates PRC2 through an allosteric mechanism, which involves H3K27me3 binding to EED and transmission of the stimulating signal to the enzyme active site (**Figure 6A**). H3K27me3 is recognized by an aromatic cage on the top face of the EED WD40 repeat (**Figure 6A**) (52). EED provides limited sequence specificity beyond the trimethylated lysine; *in vitro* binding assays indicate the A-R-Kme3-S sequence content found for H3K27me3, H3K9me3, and H1K26me3 is favored (52, 168). Similar to H3K27me3, di- and trimethylation products of a lysine residue from two accessory subunits of PRC2, JARID2 and PALI1, can also bind EED and stimulate PRC2 enzymatic activity. PRC2 stimulation by JARID2K116me3 and PALI1K1241me3 may serve as a cellular mechanism promoting H3K27me3 deposition at loci devoid of existing H3K27me3 (169, 170). The SRM (stimulation-responsive motif) of EZH2 bridges the stimulating signal to the enzyme active site through simultaneous binding to the Kme3-containing sequence positioned on the EED surface and to an otherwise partially ordered helix from the SET domain (**Figure 6A**) (138, 139). The SRM itself is a mobile structural element, adopting an ordered α-helix structure in the presence of H3K27me3 or JARID2K116me3 and becoming disordered in their absence (138, 139). Structural stabilization of the SET domain and change of structural dynamics of active site residues may both contribute to the observed allosteric enzyme stimulation (138, 139). Mutational disruption of the EED aromatic cage in mESCs compromises not only the formation of H3K27me3 chromatin domains at nucleation sites, but also the spreading of H3K27me3 from nucleation sites (171). A class of chemical inhibitors, such as A-395 and EED226, specifically targets the EED aromatic cage, which blocks H3K27me3 binding and thereby abolishes the allosteric stimulation of PRC2 (**Figure 6C**) (172, 173). These inhibitors induce tumor regression in a xenograft model and complement the anti-tumor activity of SAM-competitive inhibitors of PRC2 when acquired resistance arises (172, 173).

*Enzyme inhibition by H3K27M and EZHIP in pediatric brain tumors.* Expression of H3K27M oncohistone in diffuse midline gliomas causes global loss of the H3K27me3 histone mark (71, 72). A crystal structure captures H3K27M bound to a minimally active PRC2 (**Figure 6A**) (139). H3K27M is found to occupy the lysine binding channel at the active site of PRC2, conferring competitive enzyme inhibition by excluding the H3K27 substrate (**Figure 6A**) (139). Compared to H3K27, H3K27M displays an over 10-fold tighter binding to PRC2 (139), which also depends on the SAM concentration (174, 175). H3K27M preferentially associates with the H3K27me3-stimulated state of PRC2, leading to defects in H3K27me3 spreading while retaining H3K27me3 at PRC2 recruitment sites on selected CGIs (174–177). Remarkably, EZHIP, a protein normally found in gonads, is abnormally expressed in posterior fossa ependymoma, where it inhibits PRC2 enzymatic activity through an H3K27M-like protein sequence (73–77). Nucleosome binding to PRC2 surface helps guide residue K27 of the histone H3 tail to the enzyme active site (178, 179); likewise, additional binding sites may be needed to provide similar structural guidance to the H3K27M-like sequence of EZHIP (76).

### 6.3 Chromatin Recruitment

Whereas the core subunits of PRC2 provide critical binding surfaces to nucleosome core particles, functional domains of some accessory subunits of PRC2 are known to interact with linker DNAs and histone marks, which together dictate chromatin targeting of PRC2. Importantly, recent cryo-EM work reveals how AEBP2 and JARID2-containing PRC2.2 engages with a mononucleosome harboring the H2AK119ub histone mark (157), representing a relatively complete structural view of PRC2.2 at a functional state. In comparison, PRC2.1 in the nucleosome-bound state remains less structurally understood. The current knowledge on structural features conferring chromatin binding by PRC2 is discussed below.

**Figure 7:**
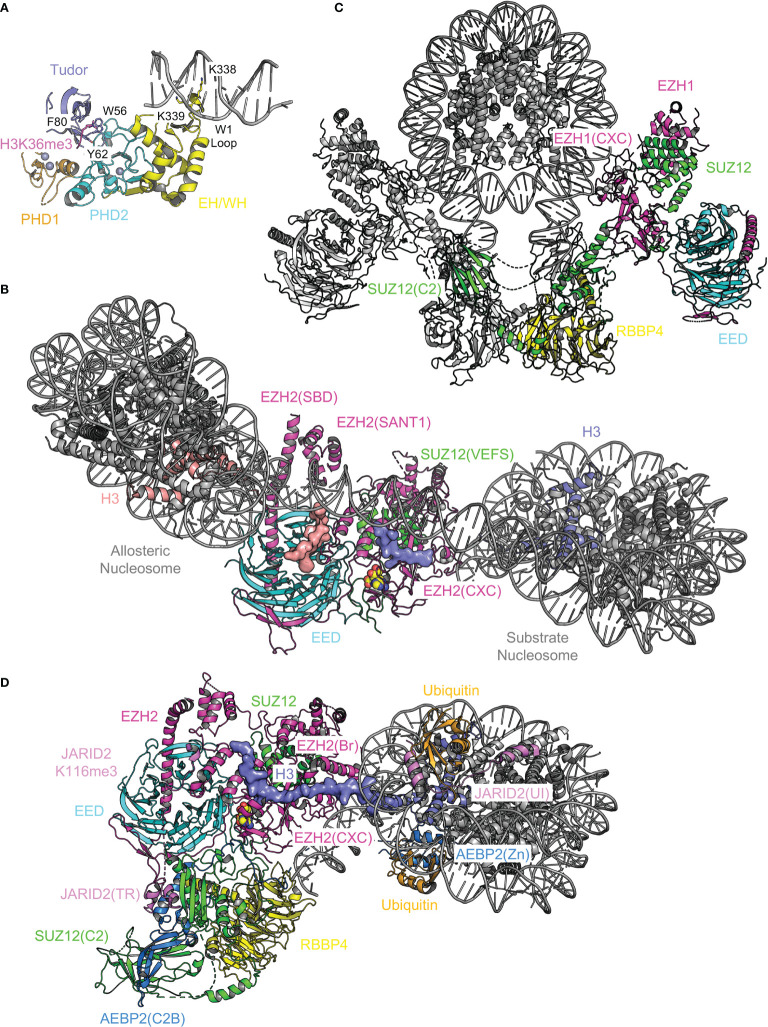
Structural analysis of chromatin binding by PRC2. Structural elements of PRC2 responsible for chromatin binding are analyzed. **(A)** Structure of MTF2(Tudor–PHD1–PHD2–EH/WH) bound to CpG DNA and H3K36me3 (PDB 5XFR). Aromatic cage residues of the Tudor domain are shown as sticks and labeled. Two lysine residues of the W1 loop of the EH/WH domain inserted into the DNA major groove are shown as sticks and labeled. **(B)** Structure of PRC2 bound to a dinucleosome (PDB not available). Only the catalytic module is visible. Substrate and allosteric nucleosomes are labeled. Two major docking sites for nucleosomes, EZH2(SBD)–EZH2(SANT1) and EZH2(CXC), are indicated. Histone H3 tail bound to the active site is shown as surface representation. **(C)** Structure of dimeric EZH1-containing PRC2 bound to a mononucleosome (PDBs 7KTQ, 7KSR, and 7KTP). Subunits of one of the two PRC2 protomers are color-coded. **(D)** Structure of AEBP2 and JARID2-containing PRC2.2 bound to a mononucleosome with H2AK119ub (PDB 6WKR). Functional domains are color-coded and labeled. The entire histone H3 tail is ordered and shown as surface representation.

*H3K36me3 and linker DNA binding by PHF1, MTF2, and PHF19.* H3K36me3 is specifically recognized by PHF1, MTF2, and PHF19, with the trimethylated lysine accommodated in an aromatic cage of the Tudor domain, which links PRC2 to chromatin to possibly mediate *de novo* silencing of active genes (**Figure 7A**) (154, 180–182). Disruption of the H3K36me3-Tudor interaction in PHF19 and PHF1 hampers PRC2 recruitment during mESC differentiation and PHF1-mediated DNA damage response, respectively (154, 180–182). Residue H3K36 is found close to the binding interface between EZH2 and nucleosomal DNAs in a recent cryo-EM structure, where H3K36 methylation is predicted to impair PRC2 enzymatic activity allosterically (179, 183). How the Tudor domain of PHF1 may contribute to H3K36me3-mediated PRC2 inhibition remains to be elucidated (181). The EH/WH (extended homology/winged-helix) domain of PHF1, MTF2, and PHF19 bind DNAs containing CpG dinucleotides, which directly facilitates PRC2 targeting to CGI chromatin in mESCs (**Figure 7A**) (184). Specifically, two lysine residues from the conserved W1 loop of the EH/WH domain of PHF1 and MTF2 are inserted into the major groove of DNA, reading the unmethylated CpG sequence content (**Figure 7A**) (184). In contrast, the EH/WH domain of *Drosophila* PCL does not seem to display sequence specificity towards DNA binding (185).

*PRC2 binding to dinucleosomes and mononucleosome binding to the PRC2 dimer.* Bifunctional dinucleosomes, in which a ‘substrate nucleosome’ is connected to a trimethyllysine analog-containing ‘allosteric nucleosome’ *via* a 30 base pair (bp), 35 bp, or 40 bp linker DNA, are captured mostly engaged with the catalytic module of PRC2 (**Figure 7B**) (178, 185). Different linker DNA lengths are accommodated by variations of the allosteric nucleosome conformation and linker DNA path, whereas substrate nucleosomes stay relatively static (178). In addition to EED aromatic cage, a major binding surface for the allosteric nucleosome is the SBD–SANT1 intramolecular complex; the substrate nucleosome contacts the CXC domain of EZH2 such that H3K27 is brought to the substrate-binding groove at the active site (**Figure 7B**) (178, 185). The accessory subunit-binding module of PRC2 also appears to loosely associate with nucleosomes (178), although its structure is difficult to resolve (178, 185). In another cryo-EM structure, an EZH1-containing PRC2 dimer is found to engage with opposite sides of a mononucleosome *via* similar interacting surfaces used by the substrate nucleosome mentioned above (**Figure 7C**) (186). The linker DNA exiting the mononucleosome also contacts the accessory subunit-binding module, through which the PRC2 dimer is formed (**Figure 7C**) (142, 186). In line with its important role in CGI DNA binding (142), PRC2 dimerization is also required for chromatin compaction by EZH1-containing PRC2 (186). Interestingly, AEBP2 and JARID2 present in the reconstituted holo complex are visible only in the structure of a monomeric form of PRC2.2 but not in the structure of the PRC2 dimer (186), which is consistent with the previous observation that AEBP2 binding disrupts the intrinsic PRC2 dimer (142).

*PRC2.2 binding to nucleosomes bearing H2AK119ub.* In contrast to the traditional hierarchical recruitment model, in which H3K27me3 added by PRC2 helps recruit PRC1 (112), recent studies indicate that H2AK119ub deposited by PRC1 can be critical for PRC2 recruitment by associating with AEBP2 and JARID2 (187–190). This model is directly supported by the cryo-EM structure of a PRC2.2 holo complex bound to a mononucleosome with chemically installed H2AK119ub (**Figure 7D**) (157). Notably, the ubiquitin interaction (UI) motif of JARID2 [JARID2(UI)] and zinc finger (Zn) domain of AEBP2 [AEBP2(Zn)] are shown to independently interact with the two copies of ubiquitin on the nucleosome (**Figure 7D**) (157). A basic bridge (Br) helix of EZH2 [EZH2(Br)] is induced to form in this context, bridging nucleosomal DNA and histone H3 tail to the SET domain (**Figure 7D**) (157). The bridge helix is also known to be disordered in the absence of the nucleosome and become automethylated in the presence of SAM, leading to PRC2 activation (191, 192).

## 7 Concluding Remarks

Recent years have witnessed substantial progress in understanding the molecular basis of gene regulation by PRC2, which also sheds light on relevant disease mechanisms and facilitates the development of mechanism-based therapeutics. The number of accessory subunits of PRC2 holo complexes is growing, new modes of action of PRC2 are constantly revealed, and general principles on the role of PRC2 in gene regulation in distinct cellular contexts have started to emerge. Some central mechanistic questions remain largely unanswered, such as those concerning the molecular interplay between PRC2 and active transcription machineries on promoters and enhancers, the architectural or orchestrating role of PRC2 in the formation of high-order chromatin structure, and the molecular nature and functional consequence of the widespread PRC2–RNA interaction in cells. Some aspects of PRC2 research are only briefly mentioned here due to limited space but are no less important, such as posttranslational modification of PRC2 components and metabolic regulation of PRC2 activity. Finally, the structural biology of PRC2 captured in various functional states will continue to make critical contributions to the field by connecting molecular structure to function.

## Author Contributions

XL (1^st^ author) and XL (2^nd^ author) wrote the manuscript. All authors contributed to the article and approved the submitted version. 

## Funding

The work in the Liu laboratory was supported by Welch Foundation research grant I-1790 and NIH grants GM121662 and GM136308. Xin Liu is a W. W. Caruth, Jr. Scholar in Biomedical Research at UT Southwestern Medical Center.

## Conflict of Interest

The authors declare that the research was conducted in the absence of any commercial or financial relationships that could be construed as a potential conflict of interest.

## Publisher’s Note

All claims expressed in this article are solely those of the authors and do not necessarily represent those of their affiliated organizations, or those of the publisher, the editors and the reviewers. Any product that may be evaluated in this article, or claim that may be made by its manufacturer, is not guaranteed or endorsed by the publisher.
